# Supplementary material for: Smad7 Binds Differently to Individual and Tandem WW3 and WW4 Domains of WWP2 Ubiquitin Ligase Isoforms
Source: Int J Mol Sci. 2019 Sep 21;20(19):4682. doi: 10.3390/ijms20194682 (PMC6801763; doi:10.3390/ijms20194682)
Supplement: Supplementary file 1 [file ijms-20-04682-s001.pdf]

Smad7 binds differently to individual and tandem WW3 and WW4 domains of WWP2 ubiquitin ligase isoforms

Lloyd C. Wahl[1,#], Jessica E. Watt[1,§], Hiu T. T. Yim[1], Danielle De Bourcier[2], James Tolchard[2,¶], Surinder M. Soond[1,✦], Tharin M. A. Blumenschein[2*], and Andrew Chantry[1*]

[1] From the School of Biological Sciences, University of East Anglia, Norwich NR4 7TJ, UK
[2] From the School of Chemistry, University of East Anglia, Norwich NR4 7TJ, UK
[#] Present address: Department of Infectious Diseases and Public Health, Jockey Club College of Veterinary Medicine and Life Sciences, City University of Hong Kong, Hong Kong
[§] Present Address: Northern Institute for Cancer Research, Newcastle University, Newcastle upon Tyne NE2 4HH, UK
[¶] Present Address: EMBL-EBI, Wellcome Genome Campus, Hinxton, Cambridgeshire, CB10 1SD, UK.
[✦] Present Address: Institute of Molecular Medicine, Sechenov First Moscow State Medical University, Trubetskaya str. 8-2, 119991, Moscow, Russian Federation.
* Correspondence: a.chantry@uea.ac.uk
* Correspondence: t.blumenschein@uea.ac.uk

Keywords: Smad, E3 ubiquitin ligase, WW domain, Smad7, TGFβ signalling, NEDD4, protein interaction

---

Supplementary Material

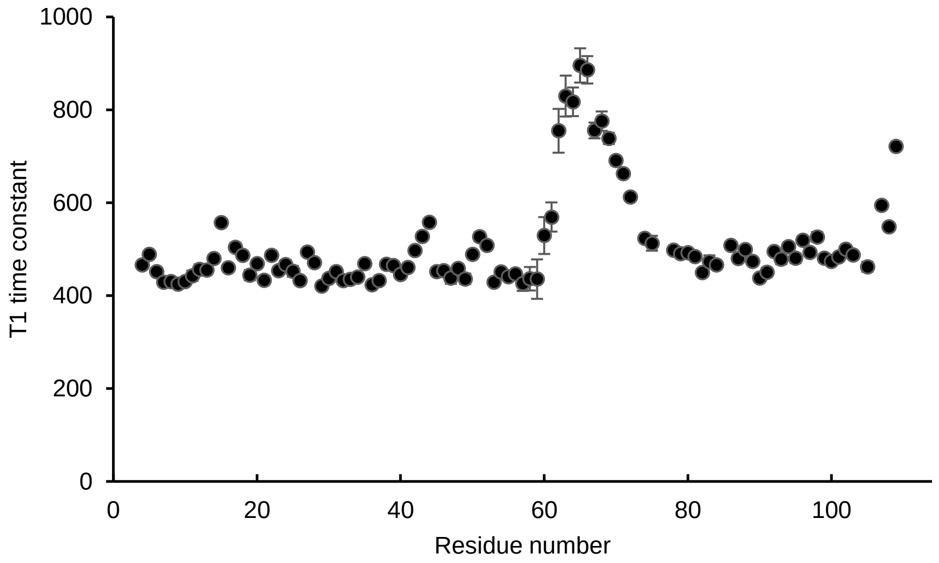

Figure S1. (**A**) Backbone amide T_1 relaxation constants for residues of the GB1-WW4 construct. GB1: residues 1-61, Linker: residues 62-66, WW4: residues 67-114.

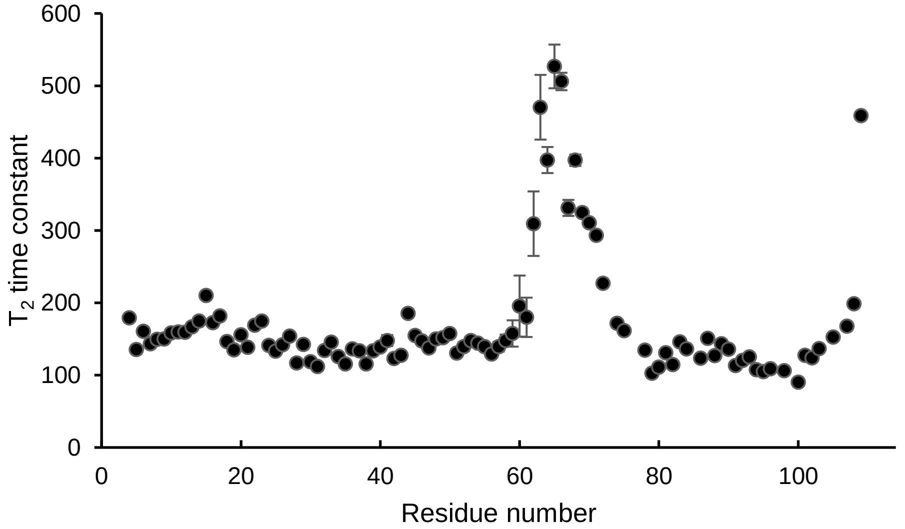

Figure S1. (**B**) Backbone amide T_2 relaxation constants for residues of the GB1-WW4 construct. GB1: residues 1-61, Linker: residues 62-66, WW4: residues 67-114.

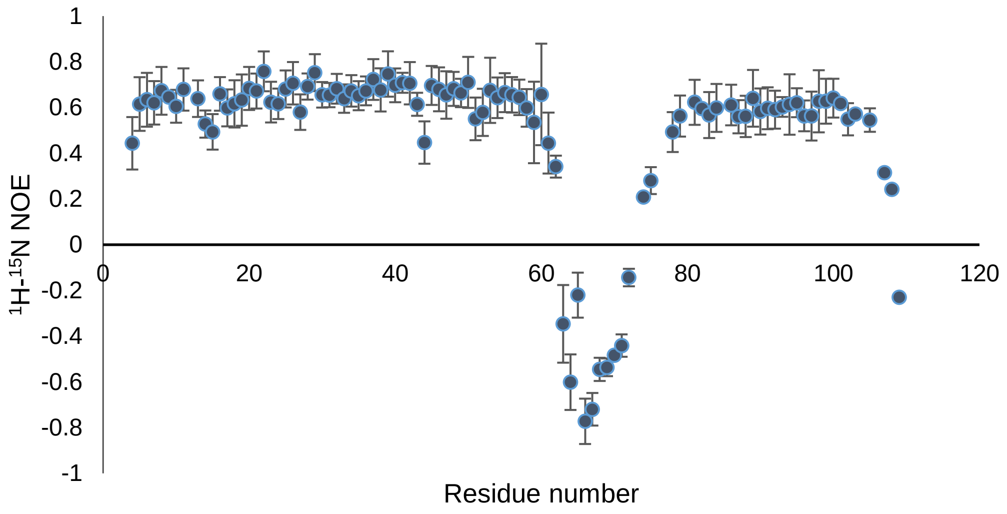

Figure S1. (**C**) Backbone amide ^{1}H-^{15}N NOE relaxation for residues of the GB1-WW4 construct. GB1: residues 1-66, Linker: residues 62-66, WW4: residues 67-114.

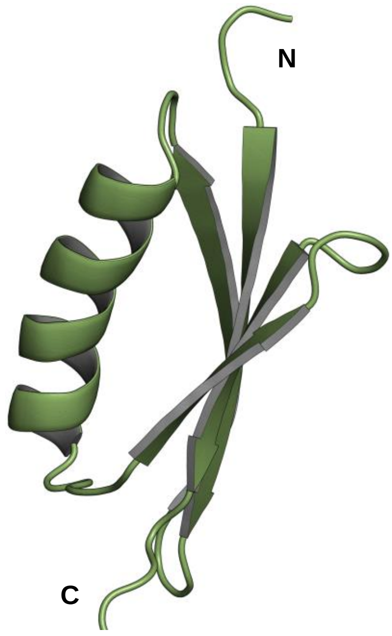

Figure S2 (**A**) Ribbon diagram depiction of the GB1 tag from the most representative model (model 16) of the refined 20-model structural ensemble.

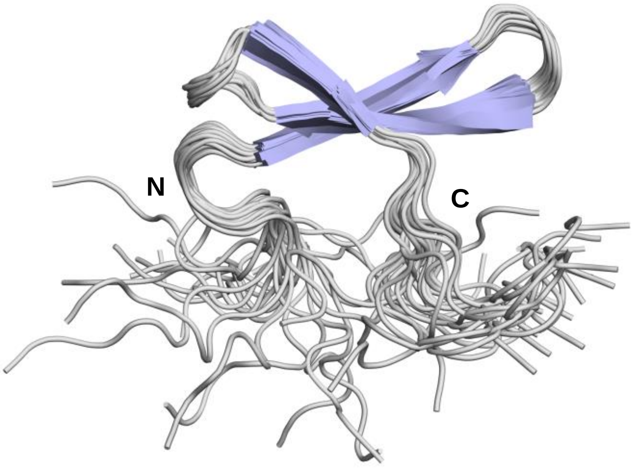

Figure S2. (**B**) Ribbon diagram depiction of the refined 20-model WW4 domain structural ensemble.

Table S1. Clustal Omega [1] alignment of NEDD4 WW domains. (An asterisk indicates fully conserved residues. A colon indicates residues with strongly similar properties. A full stop indicates weakly similar properties.)

	Domain sequence	Similarity (%)
**WWP2 WW4**	PALPPGWEMKYTSEGVRYFVDHNTRTTTFKDPRP	100
**WWP1 WW4**	EPLPEGWEIRYTREGVRYFVDHNTRTTTFKDPRN	79.41
**HECW2 WW2**	LELPRGWEMKHDHQGKAFFVDHNSRTTTFIDPRL	61.76
**SMURF1 WW2**	GPLPPGWEVRSTVSGRIYFVDHNNRTTQFTDPRL	61.76
**SMURF2 WW3**	GPLPPGWEIRNTATGRVYFVDHNNRTTQFTDPRL	61.76
**ITCH WW4**	KPLPEGWEMRFTVDGIPYFVDHNRRTTTYIDPRT	61.76
**NEDD4L WW3**	SFLPPGWEMRIAPNGRPFFIDHNTKTTTWEDPRL	55.88
**HECW1 WW2**	LELPRGWEIKTDQQGKSFFVDHNSRATTFIDPRI	55.88
**ITCH WW3**	GPLPPGWEKRTDSNGRVYFVNHNTRITQWEDPRS	55.88
**WWP2 WW3**	GPLPPGWEKRQD-NGRVYYVNHNTRTTQWEDPRT	54.55
**WWP1 WW3**	GPLPPGWEKRVDSTDRVYFVNHNTKTTQWEDPRT	52.94
**NEDD4 WW2**	SGLPPGWEEKQDERGRSYYVDHNSRTTTWTKPTV	52.94
**WWP2 WW2**	RPLPPGWEKRTDPRGRFYYVDHNTRTTTWQRPTA	52.94
**WWP2 WW1**	DALPAGWEQRELPNGRVYYVDHNTKTTTWERPLP	52.94
**WWP1 WW1**	ETLPSGWEQRKDPHGRTYYVDHNTRTTTWERPQP	52.94
**NEDD4 WW3**	GFLPKGWEVRHAPNGRPFFIDHNTKTTTWEDPRL	50
**ITCH WW2**	EPLPPGWERRVDNMGRIYYVDHFTRTTTWQRPTL	50
**WWP1 WW2**	QPLPPGWERRVDDRRRVYYVDHNTRTTTWQRPTM	50
**NEDD4L WW1**	PPLPPGWEEKVDNLGRTYYVNHNNRTTQWHRPSL	50
**HECW2 WW1**	EALPPNWEARIDSHGRIFYVDHVNRTTTWQRPTA	47.06
**NEDD4L WW2**	PGLPSGWEERKDAKGRTYYVNHNNRTTTWTRPIM	47.06
**HECW1 WW1**	EPLPPNWEARIDSHGRVFYVDHVNRTTTWQRPTA	44.12
**ITCH_WW1**	APLPPGWEQRVDQHGRVYYVDHVEKRTTWDRPEP	44.12
**NEDD4 WW1**	SPLPPGWEERQDILGRTYYVNHESRRTQWKRPTP	44.12
**SMURF1 WW1**	PELPEGYEQRTTVQGQVYFLHTQTGVSTWHDPRI	41.18
**SMURF2 WW2**	PDLPEGYEQRTTQQGQVYFLHTQTGVSTWHDPRV	41.18
**NEDD4L WW4**	GPLPPGWEERIHLDGRTFYIDHNSKITQWEDPRL	41.18
**NEDD4 WW4**	GPLPPGWEERTHTDGRIFYINHNIKRTQWEDPRL	38.24
**SMURF2 WW1**	NDLPDGWEERRTASGRIQYLNHITRTTQWERPTR	38.24
	** .:* : ::. : : *	

Table S2. GPS 3.0 phosphorylation prediction [2] at S206 of human Smad7.

	Position	Context	Score	Cutoff	Kinase	Prediction
Smad7	206	SRLCELESPPPPYSR	23.412	6.118	CMGC/MAPK/JNK/JNK2	YES
Smad7	206	SRLCELESPPPPYSR	17.346	7.867	CMGC/CDK/CDK5/CDK5	YES
Smad7	206	SRLCELESPPPPYSR	15.278	9.411	CMGC/CDK/CDK2/CDC28	YES
Smad7	206	SRLCELESPPPPYSR	13.243	4.61	CMGC/MAPK/ERK/Erk2	YES
Smad7	206	SRLCELESPPPPYSR	12.722	8.145	Atypical/PIKK/FRAP	YES
Smad7	206	SRLCELESPPPPYSR	12.625	10.326	CMGC/MAPK/ERK/Erk1	YES
Smad7	206	SRLCELESPPPPYSR	12.062	6.781	CMGC/MAPK/JNK/JNK3	YES
Smad7	206	SRLCELESPPPPYSR	11.898	4.168	CMGC/CDK/CDC2/CDK1	YES
Smad7	206	SRLCELESPPPPYSR	11.263	6.616	CMGC/CDK/CDK5	YES
Smad7	206	SRLCELESPPPPYSR	10.833	7.061	CMGC/CDK/CDK5/PHO85	YES
Smad7	206	SRLCELESPPPPYSR	10.667	3.833	CMGC/MAPK/ERK/FUS3	YES
Smad7	206	SRLCELESPPPPYSR	10.143	3.221	CMGC/CDK/CRK7	YES
Smad7	206	SRLCELESPPPPYSR	10.143	3.221	CMGC/CDK/CRK7/CRK7	YES
Smad7	206	SRLCELESPPPPYSR	10.03	4.309	CMGC/MAPK/p38	YES
Smad7	206	SRLCELESPPPPYSR	8.971	4.55	CMGC/MAPK	YES
Smad7	206	SRLCELESPPPPYSR	8.2	7.46	CMGC/MAPK/p38/HOG1	YES
Smad7	206	SRLCELESPPPPYSR	8.192	4.698	CMGC/CDK/CDC2	YES
Smad7	206	SRLCELESPPPPYSR	8.094	6.826	CMGC/CDK/CDK2/CDK2	YES
Smad7	206	SRLCELESPPPPYSR	8	4.95	CAMK/CAMKL/AMPK/AMPKA2	YES
Smad7	206	SRLCELESPPPPYSR	7.667	6.008	Other/Other-Unique/KIS	YES
Smad7	206	SRLCELESPPPPYSR	6.404	3.933	CMGC/CDK	YES
Smad7	206	SRLCELESPPPPYSR	6.403	4.507	CMGC/DYRK	YES
Smad7	206	SRLCELESPPPPYSR	6.333	4.25	AGC/PKA/PKACB	YES
Smad7	206	SRLCELESPPPPYSR	6.333	5.7	CMGC/MAPK/ERK/Erk4	YES
Smad7	206	SRLCELESPPPPYSR	6.333	3.483	Other/NEK/NEK11/NEK11	YES
Smad7	206	SRLCELESPPPPYSR	5.828	3.205	Atypical/PIKK/FRAP/MTOR	YES
Smad7	206	SRLCELESPPPPYSR	5.824	2.841	CMGC/GSK/GSK3A	YES
Smad7	206	SRLCELESPPPPYSR	5.741	5.425	CMGC/MAPK/p38/MAPK11	YES
Smad7	206	SRLCELESPPPPYSR	5.667	4	Other/MOS	YES
Smad7	206	SRLCELESPPPPYSR	5.667	4	Other/MOS/MOS	YES
Smad7	206	SRLCELESPPPPYSR	5.5	5.133	CK1/TTBK/TTBK1	YES
Smad7	206	SRLCELESPPPPYSR	4.706	3.307	CMGC/CDK/CDK4/CDK4	YES
Smad7	206	SRLCELESPPPPYSR	4.704	3.279	CMGC/DYRK/DYRK1	YES
Smad7	206	SRLCELESPPPPYSR	4.445	4.186	Atypical/PIKK	YES
Smad7	206	SRLCELESPPPPYSR	4.019	1.106	AGC/PDK1	YES
Smad7	206	SRLCELESPPPPYSR	3.612	2.944	CMGC/MAPK/JNK	YES
Smad7	206	SRLCELESPPPPYSR	3.331	1.667	CMGC/MAPK/ERK	YES
Smad7	206	SRLCELESPPPPYSR	2.469	0.899	AGC/PDK1/PDPK1	YES
Smad7	206	SRLCELESPPPPYSR	2.446	2.107	CMGC/MAPK/p38/MAPK14	YES
Smad7	206	SRLCELESPPPPYSR	2.137	1.892	CMGC/MAPK/JNK/JNK1	YES
Smad7	206	SRLCELESPPPPYSR	2.13	2.07	CMGC	YES
Smad7	206	SRLCELESPPPPYSR	1.704	1.499	CMGC/CDK/CDK2	YES
Smad7	206	SRLCELESPPPPYSR	1.515	0.897	CMGC/DYRK/HIPK	YES
Smad7	206	SRLCELESPPPPYSR	1.5	0.3	CMGC/MAPK/ERK/Erk3	YES
Smad7	206	SRLCELESPPPPYSR	1.379	1.199	CMGC/DYRK/HIPK/HIPK2	YES
Smad7	206	SRLCELESPPPPYSR	0.242	0.213	CMGC/CDK/CDK7/CDK7	YES
Smad7	206	SRLCELESPPPPYSR	0	0	TKL/RIPK	.

Table S3. NetPhos 3.1 phosphorylation prediction [3,4] at S206 of human Smad7.

	Position	Context	Score	Cutoff	Kinase	Prediction
Smad7	206	CELESPPPP	0.614	0.5	cdk5	YES
Smad7	206	CELESPPPP	0.519	0.5	cdc2	YES
Smad7	206	CELESPPPP	0.51	0.5	GSK3	YES
Smad7	206	CELESPPPP	0.484	0.5	p38MAPK	.
Smad7	206	CELESPPPP	0.444	0.5	CaM-II	.
Smad7	206	CELESPPPP	0.368	0.5	CKI	.
Smad7	206	CELESPPPP	0.353	0.5	DNAPK	.
Smad7	206	CELESPPPP	0.304	0.5	CKII	.
Smad7	206	CELESPPPP	0.302	0.5	ATM	.
Smad7	206	CELESPPPP	0.296	0.5	RSK	.
Smad7	206	CELESPPPP	0.287	0.5	PKG	.
Smad7	206	CELESPPPP	0.106	0.5	PKA	.
Smad7	206	CELESPPPP	0.092	0.5	PKB	.
Smad7	206	CELESPPPP	0.066	0.5	unsp	.
Smad7	206	CELESPPPP	0.053	0.5	PKC	.

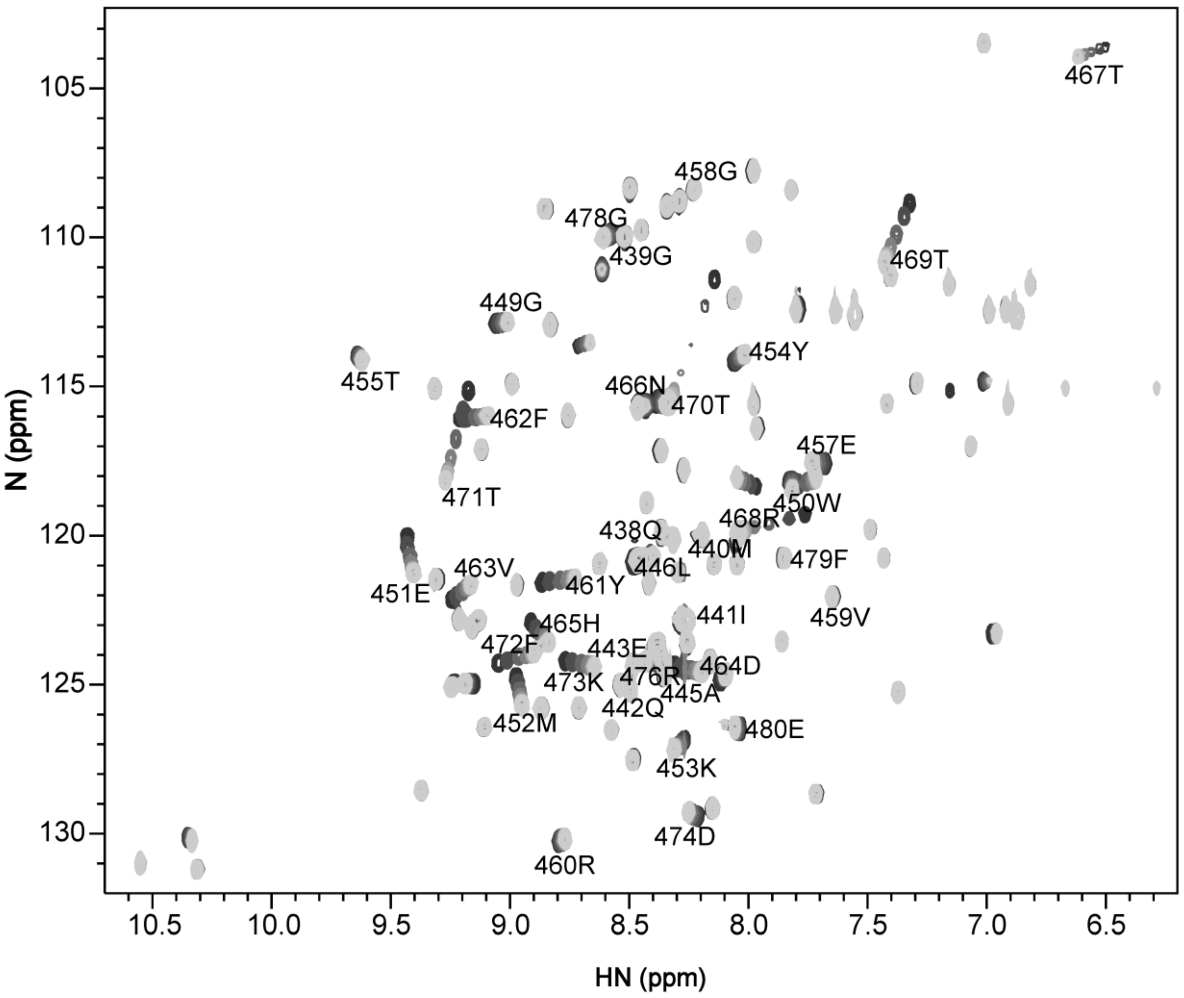

Figure S3. (**A**) The superimposed WW4/Smad7 titration HSQCs. Lower ligand concentrations are in light grey and higher ligand concentrations are in dark grey.

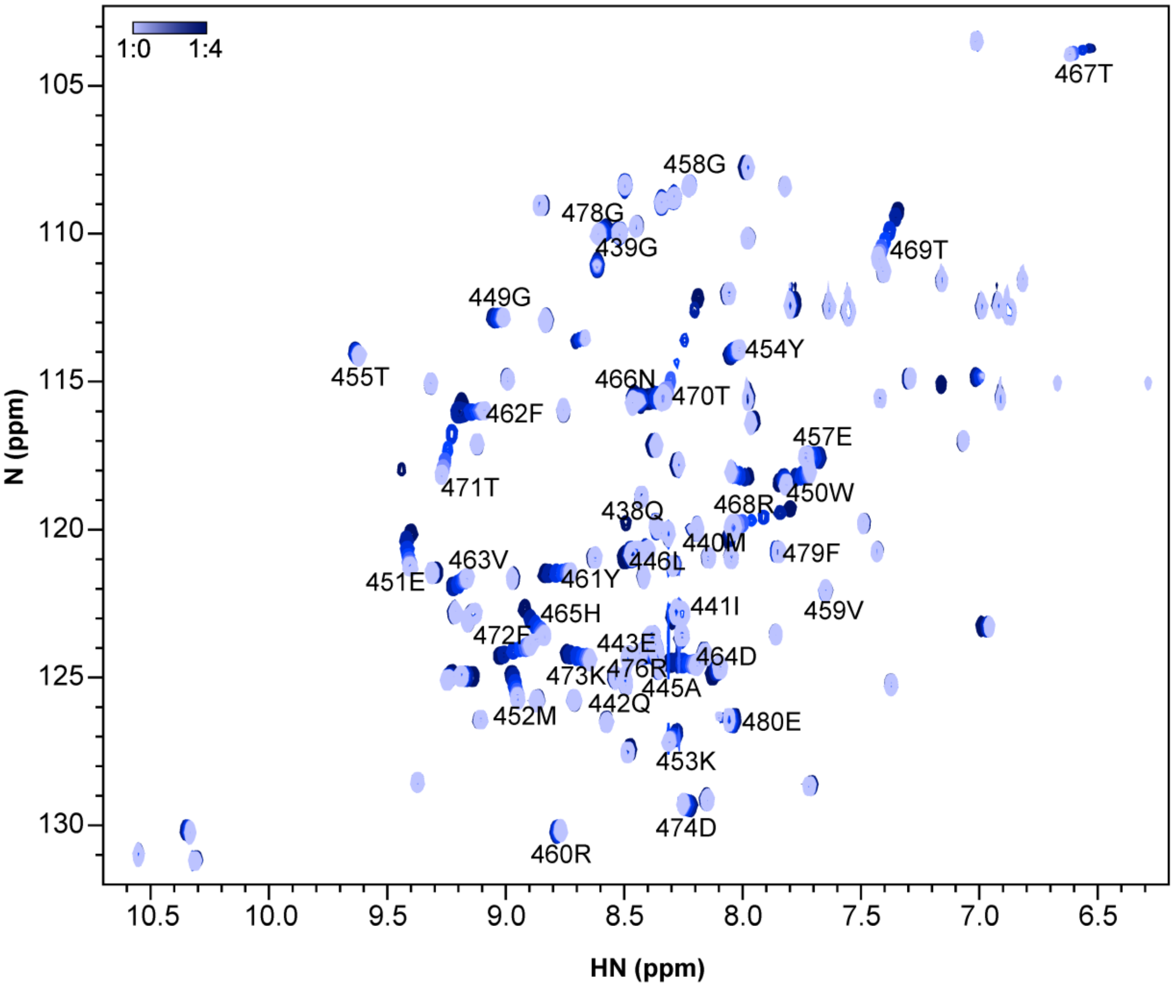

Figure S3. (**B**) The superimposed WW4/pSmad7 titration HSQCs. Lower ligand concentrations are in light blue and higher ligand concentrations are in dark blue.

5′- GCAGTCCTGTTTATGTTTCAAAACTATATACATA**TGAC**TTCG**GGCCG**GGTGCAGG**G**
GCTCACACCTGTAATCCTAGCACTTTGGGA**GGCTG**AGGCGAGAGGATTGCTTGAGCCCAG
GAGTTTGAGACTAGCCTGAGCCACACGGGGA**GGCT**TCCAT**GTCT**ACAAAAATTTTTTTAA
AAAATTAGTTGAATGTGATGGTGCATTCCTGTGGTCCTAGCTATTCAGGA**GGCT**AAGGTG
GGAGGATCACTTGAGCCTGGGAGGTGA**GGCTG**CAGTGAGCCATGATCCTGCTACTCTACT
CTAGCCTGGACTAACAGAGTGAGACCCTGTCAAAAAAAAAAAAAAATCTTTGGTAACAAA
AATAAATAAAAATCACTAGTTTAGACAAATTATGAGAAAGCTAGGAAGGTTCCAGATTTT
TGTGAGCCTTGTGTACTTACTAAAGGATTT**TGAC**TCAAGGGCAATGGGGAGTCAATGGAA
GTGTTGTTGTTGTTGTTGTTGTTGTTTGAGACAGGATCTCACTCTGTCGCCCA**GGCTG**GA
ATGTAGCAGCATAATCATAGTTC**ACTG**TAGCCTTGATCTCCCAGGCACAAGCAGTCCTCC
CACCTCAGCTGCCCAAGTAGCTGGGACCAAG**GGCGC**ACACTCCACCACCCCCAGCTAATT
TTTAAAAA**ATATTT**TTGTAGAGATGGGG**GTCT**CCCTCTGTTGCTTA**GGCTG**GTCAATTGA
AGTTTTTGAGCAGGAGTG**TGAC**CGAAG**GGCT**CA**GTCT**AGTGGGTTACTCTGAT**GGCT**ATT
TGTA**GGCTG**GATGGAGTGGGGAGGTTAATTCTGGGGTAGGAGCAGTAGTCCAGGGGTGAG
GTGATACAGATCTGGACATAGGAATGGAAAATGGAAAGTCAGTGGCAGGGAG**ACTG**AAAA
AGCAGCAGTCA**GGCTG**AATGATTGATAGCAGCGGGAATGATGTTGGCCTGGTTTCATCCC
TGGGAGGAGAAT**TGAC**ATGAGGAG**ACTG**AAGAAAGGCCTGGGTGTGGGGAGAGAGCTGTG
GCATTCAATTGAATGAAGGG**TGAC**AGCTGGTCATGAAGGTGAGGCACTCTAGCAGGCAGA
ATCCAGACAAAATCTGGGA**ACTGACTG**TGGGTCCTAGGAACCACTTGAGGGGAAACCATT
TTTAGGGAGG**CCTTGAG**TTTAAAAAAAAAATCATTGA**GGCCG**GGTGCAGT**GGCT**CACGCCT
GTAATCTCAGCACTTTGGGA**GGCCG**AGGTGGGAGGATCGTTTGAGTCCAGGAGTTCGAGA
CCA**GTCTG**GGCAACATGGCGAAACTCCATCTCTACTAAAAA**TATAAA**CAGTGAGACTCT**G**
TCTCAAAACAAAACAAAACCAAAGACCATGCAACATGTTTAC**ACTG**GCACCTTATATCAG
GTGAGTTTCAGTTGCAAGTAACAAAAGTTGTTAGCAACATT**GGCT**TAAATAAGGAAGGGA
ATGTATT**GGCT**CATGTA**ACTG**AAAATTCTAGATATCAATCCTGATTCCAAGCGAAGCTTG
GGCTACTCAACAATATCACCAAGGAATT**GGCT**TTTCTCCCATTTTTCTGCTCTGCCATCC
ACTGGGTCAACTTCCTATT**GTCT**TCGGGTCCAAAATAATAGCCAGTGGCCCCCAGGACCA
CATGCTTCCTCAGTCATATTCAGAAAGGATGAGCAACTATTATTTTTCCTAGATTTGCTA
GCAGATGTCCTGAGATT**GGCTG**T**GGCTGGCT**CAACATACATCACATGTGCACTTCTGGTC
CAGGCAACCATGCAACTCTGAGGTTGGGAACAGAGTCAGCTTCCCCAGAACCACATGGAT
CCCCAAATTAGAAATTGAGAGCTAATGGCAAGGAGGGATTGGGTGCTGCAGAGGCAACCA
CAGAT**GTCTG**CTATAATCAGCTAAGCCCCAGCAACCTCTG**TGAC**AGGGCCTGCCC**GGCTG**
TGCTAGGCATCCTGAGACTTTTCTAGAATTGGGGGATGGGAGTGGGGTGGTCATTATATT
CATTCTGAGGTTCCTATTTCCAG - 3′

Figure S4. (**A**) The nucleotide sequence of the predicted WWP2-ΔHECT promoter within intron 9/10. Putative AP-1, Smad [5] and SOX9 responsive elements highlighted in purple, cyan and yellow respectively. TATA boxes are highlighted in grey.

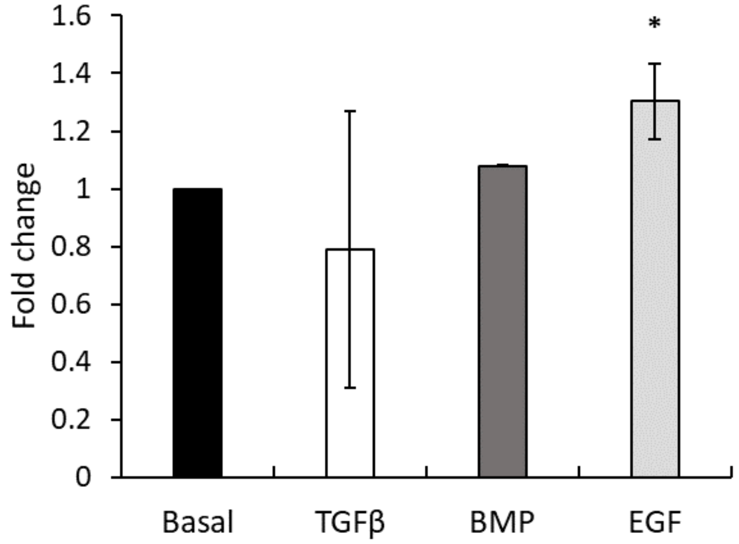

Figure S4 (**B**) Luciferase activity of the prospective WWP2-ΔHECT 2 kb promoter region under different growth factor conditions in HEK293A cells

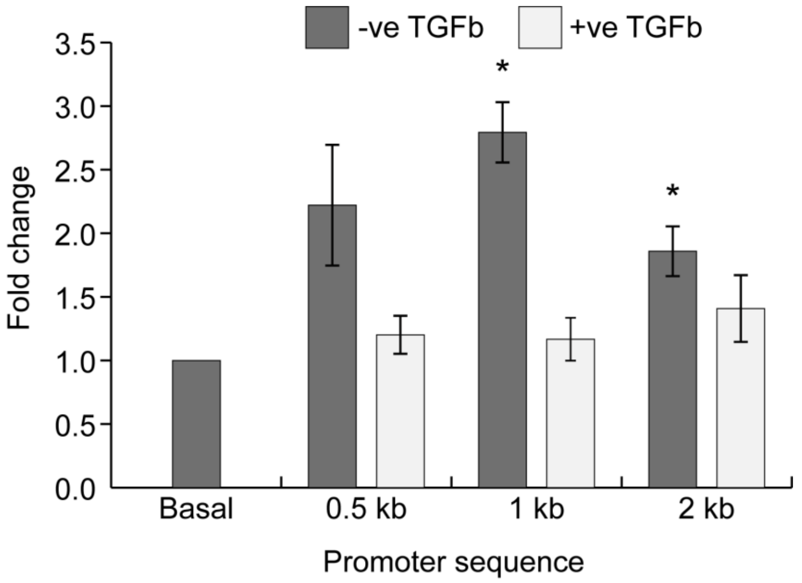

Figure S4 (**C**) Luciferase activity of different regions of the prospective WWP2-ΔHECT promoter in HEK293A cells

Table S4 – Residues in intermediate exchange in WW3/Smad7 and WW3-4/Smad7 titrations

WW3-4/Smad7	***WW3/Smad7***
410Gly	416Asp
413Lys	427Thr
416Asp	428Arg
426Asn	430Thr
427Thr	431Gln
428Arg	432Trp
430Thr	433Glu
431Gln	
432Trp	
433Glu	

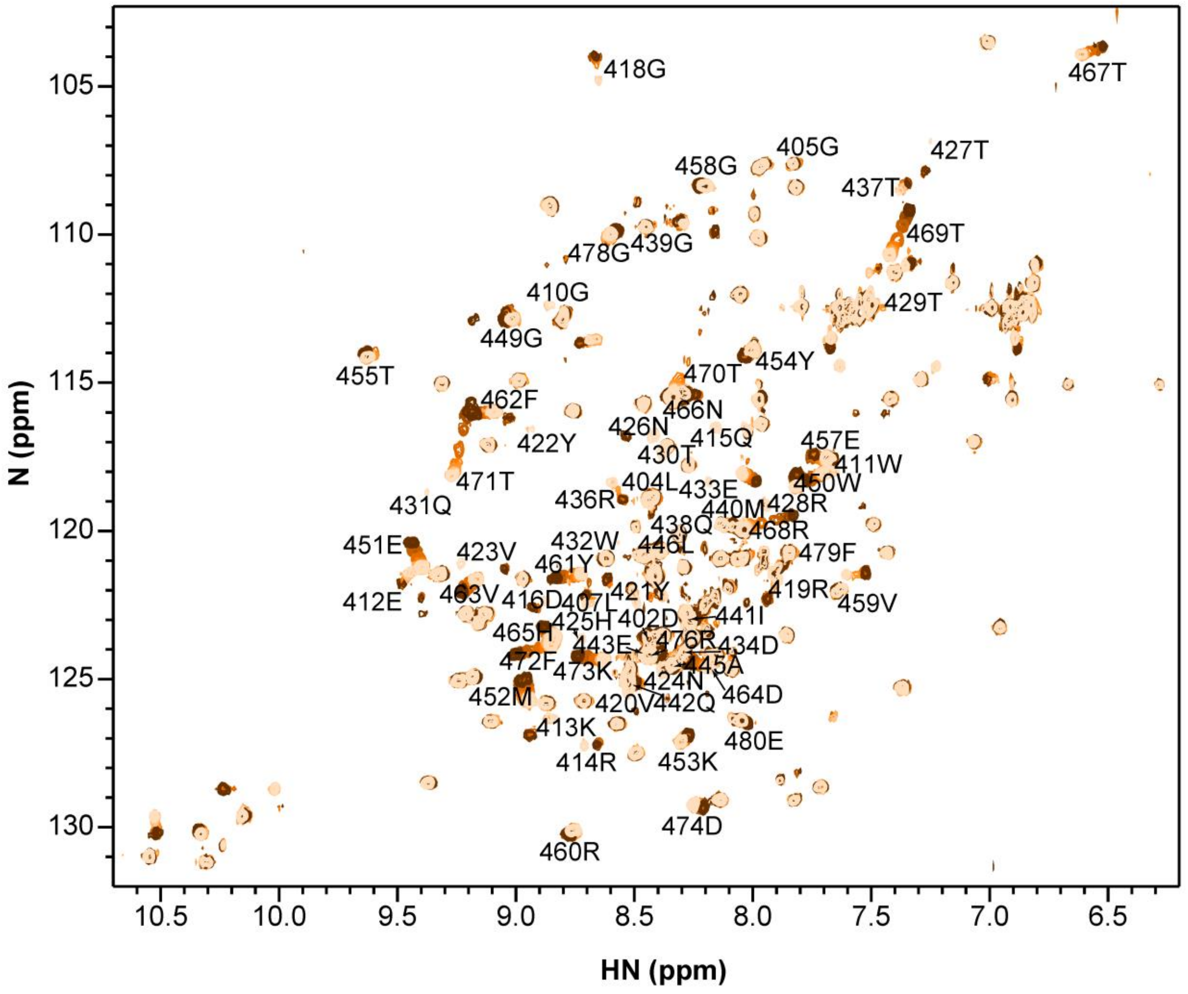

Figure S5. (**A**) The superimposed WW3-4/Smad7 titration HSQCs. Lower ligand concentrations are in light orange and higher ligand concentrations are in dark orange.

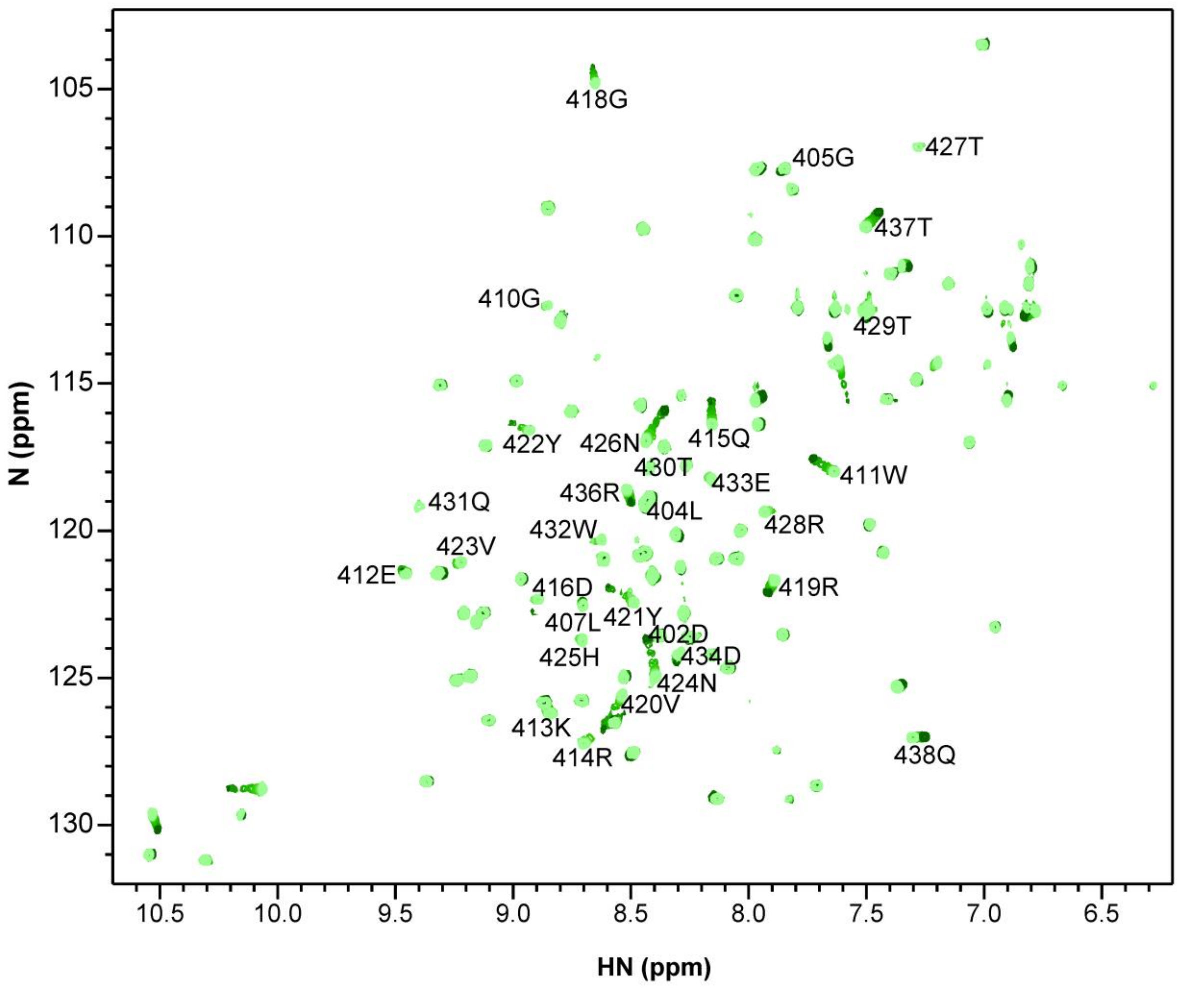

Figure S5. (**B**) The superimposed WW3/Smad7 titration HSQCs. Lower ligand concentrations are in light green and higher ligand concentrations are in dark green.

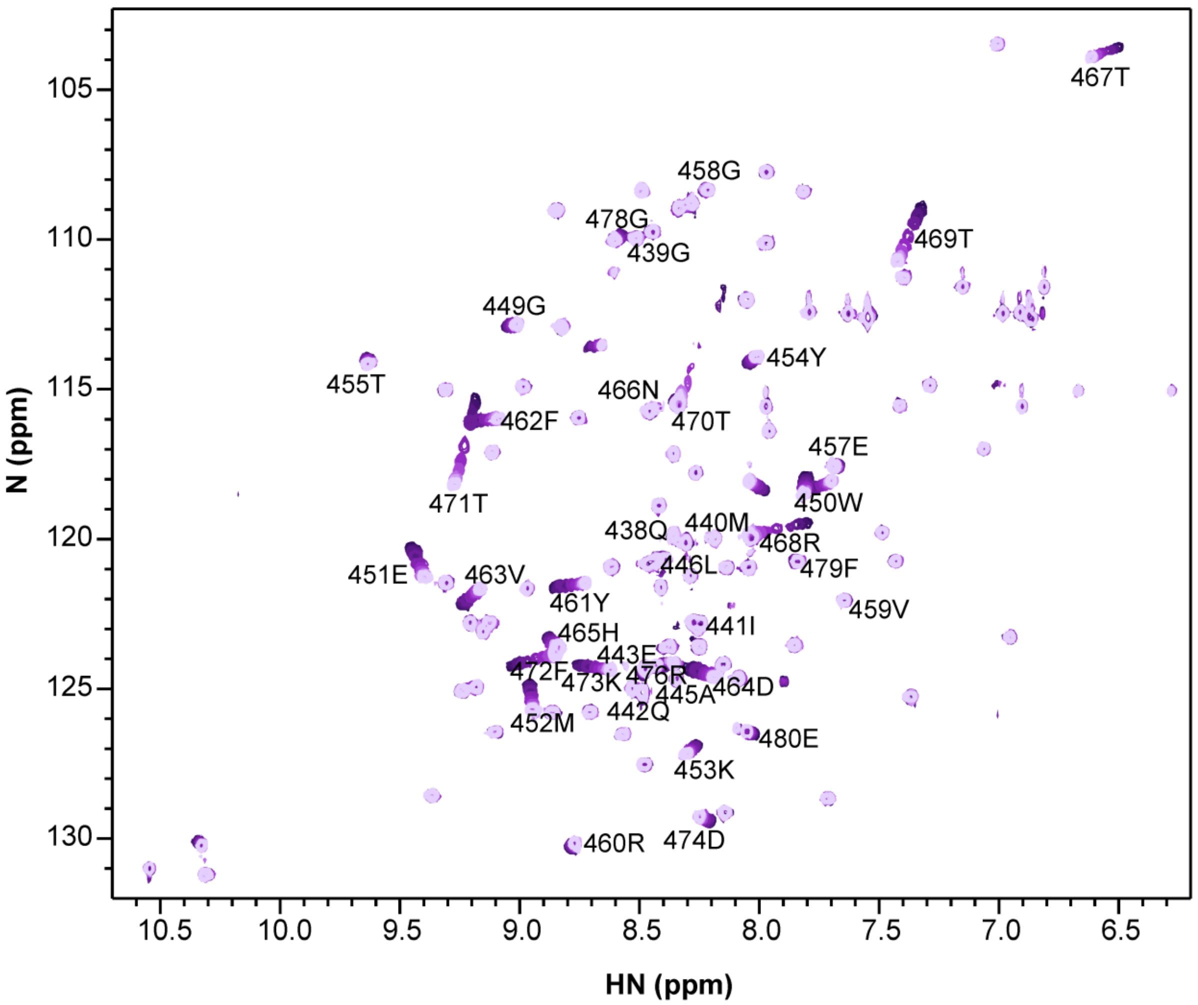

Figure S5. (**C**) The superimposed WW4/Smad7 titration HSQCs. Lower ligand concentrations are in light purple and higher ligand concentrations are in dark purple.

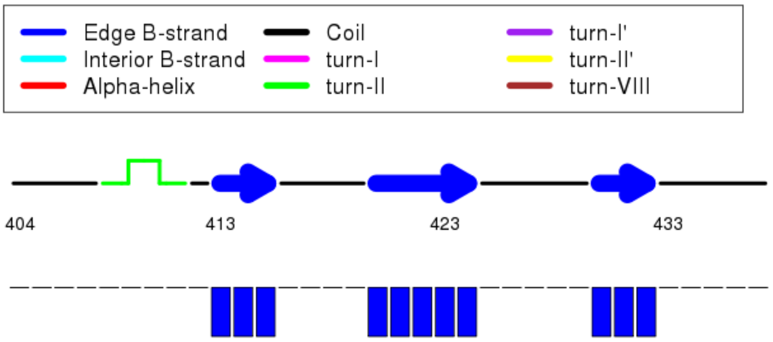

Figure S5. (**D**) The CSI 3.0 webserver [6] output predicting the secondary structure of WW3 based on backbone chemical shifts.

Table S5 – Acquisition parameters for the GB1-WW4 NMR spectra

Spectrum	Scans	Complex points			Spectral width		
		^{1}H	^{15}N	^{13}C	^{1}H	^{15}N	^{13}C
^{1}H-^{15}N-HSQC	8	1024	256	-	12019	2595	-
^{1}H-^{13}C-HSQC	64	1024	-	256	12019	-	16077
CBCA(CO)NH	24	1024	64	130	12019	2433	15105
HNCACB	24	1024	64	132	12019	2433	15105
CC(CO)NH	32	1024	42	128	12019	2433	15105
H(CCO)NH	48	1024	48	160 (^{1}H)	12019	2433	11990 (^{1}H)
HCCH-TOCSY	8	1024	120 (^{1}H)	32	7507.5	6995.6 (^{1}H)	9423.8
CCH-TOCSY	8	1024	120 (^{13}C)	32	7507.5	9423.8 (^{13}C)	9423.8
HbCbCgCdHd	256	1024	-	48	12820.5	-	6038.6
Aromatic ^{13}C TOCSY	32	1024	-	48	11160	-	2823
Aromatic TROSY HSQC	1024	1024	-	256	7212	-	8052
Aromatic NOESY[1]	16	1024	256 (^{1}H)	0	12019	10395 (^{1}H)	15105.7
^{15}N-NOESY-HSQC	32	1024	48	152(^{1}H)	7508	1519	6997 (^{1}H)
^{13}C-NOESY-HSQC	16	1024	192 (^{1}H)	64	11161	10395 (^{1}H)	15105

[1](1H-1H 2D plane NOESY-HSQC @ 120 ppm for 13C)

Supplementary Methods

NMR Spectroscopy

^{15}N T_1, ^{15}N T_2, and ^{1}H-^{15}N NOE backbone amide relaxation experiments [7] were acquired at 293 K with a Bruker Avance I 500 MHz NMR spectrometers and ^{15}N-labelled GB1-WW4. T_1 measurements were performed with a 5 s recovery delay and relaxation delays of 0.02, 0.1, 0.2, 0.5, 0.75, 1, 2, and 4 s. The relaxation delays of 0.02, 0.5, and 1 s were repeated to evaluate data consistency. T_2 measurements were performed with a 5 s recovery delay and relaxation delays of 18, 53, 88, 141, 176, 211 and 263 ms. Relaxation delays of 18, 88 and 176 ms were repeated to evaluate data consistency. NOE measurements used a saturation delay of 5 s, replaced by a relaxation delay of 5 s in the reference experiment. NMR data were processed and analysed as described in the main text.

Luciferase assay

Constructs were made by cloning 2 kb, 1 kb or 0.5 kb of the same region of *wwp2* intron 19/20 into the pGL4.27 vector (Promega). HEK293A cells were transfected with either of these plasmids and the pSV-β-Galactosidase control vector (Promega). Cells were stimulated with TGFβ (5 ng ml^{-1}), BMP-4 (10 ng ml^{-1}) or EGF (10 ng ml^{-1}) and incubated overnight at 37˚C, 5% CO_2. Luciferase activity was determined using the Promega Luciferase Assay System following the manufacturer's instructions. Data were normalised against β-galactosidase activity.

Supplementary References

1. Sievers, F.; Wilm, A.; Dineen, D.; Gibson, T.J.; Karplus, K.; Li, W.; Lopez, R.; McWilliam, H.; Remmert, M.; Söding, J.; et al. Fast, scalable generation of high-quality protein multiple sequence alignments using Clustal Omega. *Mol. Syst. Biol.* **2011**, *7*, 539.

2. Xue, Y.; Ren, J.; Gao, X.; Jin, C.; Wen, L.; Yao, X. GPS 2.0, a Tool to Predict Kinase-specific Phosphorylation Sites in Hierarchy. *Mol. Cell. Proteomics* **2008**, *7*, 1598–1608.

3. Blom, N.; Sicheritz-Pontén, T.; Gupta, R.; Gammeltoft, S.; Brunak, S. Prediction of post-translational glycosylation and phosphorylation of proteins from the amino acid sequence. *Proteomics* **2004**, *4*, 1633–1649.

4. Blom, N.; Gammeltoft, S.; Brunak, S. Sequence and structure-based prediction of eukaryotic protein phosphorylation sites. *J. Mol. Biol.* **1999**, *294*, 1351–1362.

5. Martin-Malpartida, P.; Batet, M.; Kaczmarska, Z.; Freier, R.; Gomes, T.; Aragón, E.; Zou, Y.; Wang, Q.; Xi, Q.; Ruiz, L.; et al. Structural basis for genome wide recognition of 5-bp GC motifs by SMAD transcription factors. *Nat. Commun.* **2017**, *8*, 2070.

6. Hafsa, N.E.; Arndt, D.; Wishart, D.S. CSI 3.0: a web server for identifying secondary and super-secondary structure in proteins using NMR chemical shifts. *Nucleic Acids Res.* **2015**, *43*, W370–W377.

7. Farrow, N.A.; Muhandiram, R.; Singer, A.U.; Pascal, S.M.; Kay, C.M.; Gish, G.; Shoelson, S.E.; Pawson, T.; Forman-Kay, J.D.; Kay, L.E. Backbone Dynamics of a Free and a Phosphopeptide-Complexed Src Homology 2 Domain Studied by 15N NMR Relaxation. *Biochemistry* **1994**, *33*, 5984–6003.
